# Fast Field Calibration of MIMU Based on the Powell Algorithm

**DOI:** 10.3390/s140916062

**Published:** 2014-08-29

**Authors:** Lin Ma, Wanwan Chen, Bin Li, Zheng You, Zhigang Chen

**Affiliations:** 1 Department of Precision Instrument, Tsinghua University, Beijing 100084, China; E-Mails: wwchenrun@163.com (W.C.); libin@mail.tsinghua.edu.cn (B.L.); yz-dpi@mail.tsinghua.edu.cn (Z.Y.); 2 State Key Laboratory of Precision Measurement Technology and Instruments, Tsinghua University, Beijing 100084, China; 3 National Defense Key Laboratory of Underground Damage Technology, North University of China, Taiyuan 030051, China; E-Mail: czg@nuc.edu.cn

**Keywords:** MIMU, multi-position calibration, compensation, navigation

## Abstract

The calibration of micro inertial measurement units is important in ensuring the precision of navigation systems, which are equipped with microelectromechanical system sensors that suffer from various errors. However, traditional calibration methods cannot meet the demand for fast field calibration. This paper presents a fast field calibration method based on the Powell algorithm. As the key points of this calibration, the norm of the accelerometer measurement vector is equal to the gravity magnitude, and the norm of the gyro measurement vector is equal to the rotational velocity inputs. To resolve the error parameters by judging the convergence of the nonlinear equations, the Powell algorithm is applied by establishing a mathematical error model of the novel calibration. All parameters can then be obtained in this manner. A comparison of the proposed method with the traditional calibration method through navigation tests shows the classic performance of the proposed calibration method. The proposed calibration method also saves more time compared with the traditional calibration method.

## 1. Introduction

Inertia technology includes inertia sensors, inertial navigation, inertial guidance, inertial measurement, and inertial stability. This technology is a core source of modern precision information navigation, guidance, and control systems. The microelectromechanical system (MEMS) inertial technology is a set of precision instruments, precision machinery, micro electronics, and semiconductor integrated circuit process technology and has, thus, become one of the world's most progressive technologies; inertial technology has emerged as an important research direction in recent years [1]. As a novel type of a strapdown inertial navigation system (SINS), micro inertial navigation has inherited almost all merits of the traditional SINS: completely autonomous; highly secure, which is very important in military applications; free of electromagnetic interference; and available and flexible under all weather conditions. It also has the following unparalleled advantages over the traditional SINS: small size, light weight, low cost, low power consumption, long lifetime, high reliability, wide dynamic range, fast response, and ease of installation and commissioning. Therefore, micro inertial measurement unit (MIMU) has become a hot research topic [2–4] and an important direction of the development of inertial technology for commercial and military fields [5].

Generally, one MIMU consists of accelerometers, gyroscopes, and sometimes magnetometers whose sensitive axes are orthogonally mounted to measure accelerations, angular velocities, and attitudes. These components are numerically integrated further for position estimation, which has become central to inertial guidance and control systems for missiles, rockets, and other aircraft. However, given the characteristics of MEMS materials, micro inertial sensor errors have become a major factor in determining the accuracy of inertial navigation systems. Such errors can be addressed through calibration based on error models. The main error sources of MIMU involve zero bias, scale factors, and misalignments [6]. Experiments have shown that the effects of the bias error of micro accelerometers and gyroscopes on navigation are quadratic and cubic with the growth of time [7]. MIMU calibration is a process of determining the coefficients that transform the raw outputs of inertial sensors to meaningful quantities of interest [8]. Traditional calibration tests often require the use of special references, such as alignment to a given frame, or specialized equipment, such as high-precision two-axis or three-axis turntables in laboratories. The 12-position static calibration and rate tests are among the most commonly used calibration methods [9]. The basic principle of the 12-position static test method is the use of directional and horizontal references provided by a turntable and the setting of the Earth's rotation angular velocity and acceleration of gravity as the MIMU nominal input. The accelerometer and gyroscope output are compared with the nominal input in each position after rotating the turntable into multiple locations. The least squares method is used in accordance with the equations of both the accelerometer and the gyro error model to determine the error parameters. However, the static test is useless for MEMS gyros that are blind to the Earth's reference signal as a result of the noise level. Rate tests are similar to the 12-position static test method that can be conducted by rotating the MIMU through a given angular rate and comparing the gyro outputs with these references. By combining these two methods, almost all error parameters of the MIMU can be determined.

Given the characteristics of MEMS devices, bias, scale factor, and misalignment drift with time occur every time the power is turned on and off, especially for the very sensitive bias. The practical application environment of the MIMU is different from the laboratory calibration environment, that is, the error parameters in the real application and calibration results are inconsistent. However, the difference can contribute to the accumulation of errors that lower the inertial navigation accuracy. High cost, critical turntable requirements, long calibration time, method complexity, and heavy calibration workload are the drawbacks of traditional calibration methods. These conditions limit the wide use of traditional calibration methods in key areas, such as in the battlefield. A new, low-cost, and convenient calibration method is thus needed to obtain the error parameters of the MIMU. In recent years, the multi-position calibration method has been proposed. As the fundamental principles of this method, the norm of the accelerometer measurement vector is equal to the gravity magnitude, and the norm of the gyro measurement vector is equal to the rotation rate [10–17]. Unlike in traditional calibration methods, the IMU in the multi-position calibration method need not be aligned to the local level frame, and only a single-axis turntable is used to provide an angular rate for the calibration of the MEMS gyroscope. The total calibration time and costs thus decrease, and the method can meet the demand for fast field calibration.

The Powell fitting method is used in this study to calculate the error parameters of the MIMU by solving the nonlinear equations of the multi-position calibration method. The structure of this article is as follows. Sections 2 and 3 describe the main mechanism of traditional calibration methods and the fast multi-position calibration method based on the established MIMU error model. Section 4 presents an analysis of the results of the different methods by comparing the traditional and multi-position calibration methods in a laboratory. Section 5 reports the experimental basis of the multi-position calibration results to verify the correctness of the multi-position calibration. Finally, Section 6 presents the summary and discussion.

## 2. Sensor Error Models and Traditional Calibrations of MIMU

As mentioned above, a MIMU is generally composed of orthogonal MEMS accelerometers and orthogonal MEMS gyroscopes. Error models are established based on the formation of error sources, which include biases, scale factors, random walks, and noise of MEMS sensors [18]. The error model for MEMS accelerometers can be expressed as follows:

(1) $$l_{a}=a+b_{a}+S_{1}a+S_{2}a^{2}+Na+\delta g+ɛ(a)$$

where *l_a_* is the measured acceleration of the MIMU accelerometer; *a* is the set of true MIMU inputs; *b_a_* is the accelerometer bias; *S*_1_ and *S*_2_ are the linear and non-linear scaling factor matrices, respectively; *N* refers to the misalignment matrix among the accelerometer axes; δ*g* denotes the error term associated with the acceleration of gravity; and ε(*a*) represents the output noise of each axis in the accelerometer. Similarly, the MEMS gyro error model can be expressed as follows:

(2) $$l_{\omega }=\omega +b_{\omega }+s\omega +N\omega +ɛ(\omega )$$

where *l*_ω_ is the measured angular rate, ω is the true input angular rate, *b*_ω_ represents the gyro bias, *S* is the matrix of scaling factors, and *N* is the misalignment of different gyro axes.

As indicated by the two equations above, bias, scale factor, and non-orthogonality errors are the deterministic elements and the main results calibrated by most calibration methods [13]. If sensor errors are uncompensated, these factors quickly reduce the accuracy of control and navigation systems. Thus, calibrations must be performed prior to the use of the MIMU to determine error parameters and to ensure compensation in the actual navigation.

### 2.1. Analysis of Allan Variance

**Figure 1.**
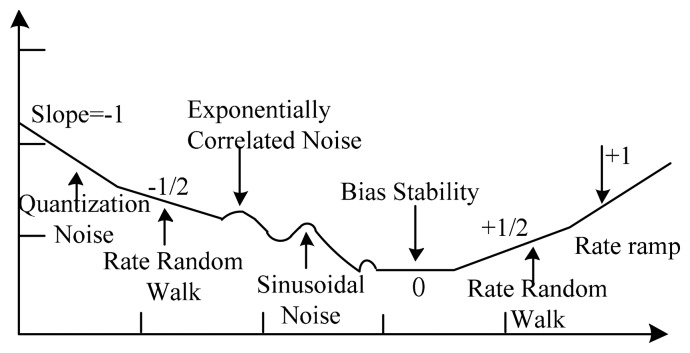
Sample plot of Root Allan variance (RAV) analysis results [24].

Several measurements have been devised for the stochastic modeling of the random errors of the MIMU. The Allan variance (AV) is the most prevalent method that is used to determine the characteristics of underlying random processes that produce data noise [19,20]. The AV method does not only identify multiple error terms but also draws excellent error separation [21]. This technique can be used to characterize various types of error terms in the inertial sensor data by performing certain operations on the entire data length [22]. The basic principle is to separate each random error term coefficient, including the angular rate random walk, bias instability, rate random walk, and quantization noise, from the log–log curve of the root mean square deviation of the output as a function of the average time [23]. The core formula is expressed as follows:

(3) $$\sigma^{2}(\tau )\frac{1}{2\tau^{2}(N-2m)}\sum_{k=1}^{N-2m}{\left(\theta_{k+2m}-2\theta_{k+m}+\theta_{k}\right)}^{2}$$

where *N* is the number of total data points in the entire run, *m* is the number of data points contained in one cluster, τ_0_ is the sampling time with a cluster time τ=*m*τ_0_, and θ is the MIMU output. The Root Allan variance (RAV) is used to plot figures. A typical RAV plot is similar to the one shown in Figure 1.

**Figure 2.**
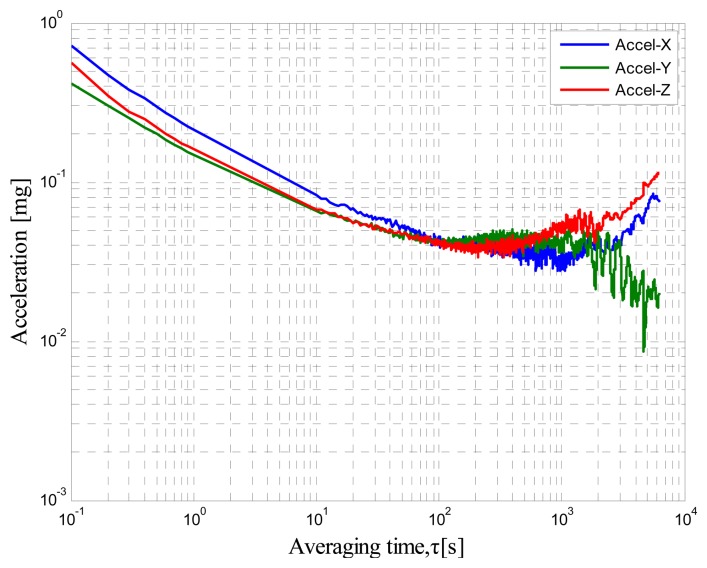
Accelerometer RAV results.

**Figure 3.**
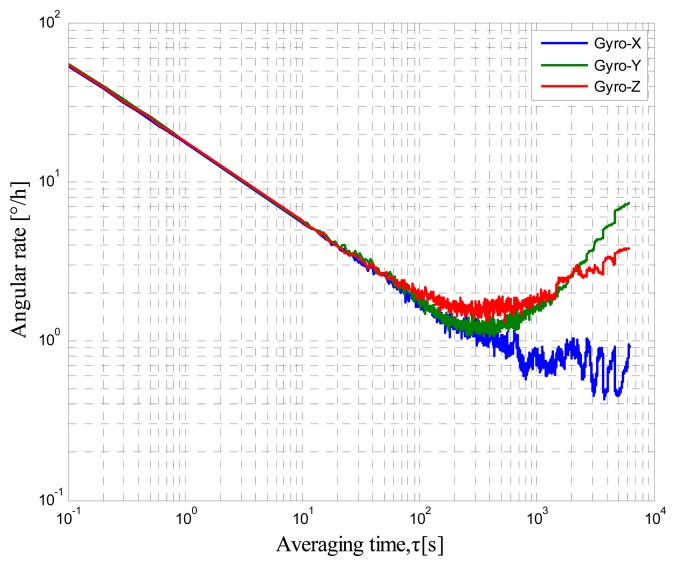
Gyro RAV results.

**Figure 4.**
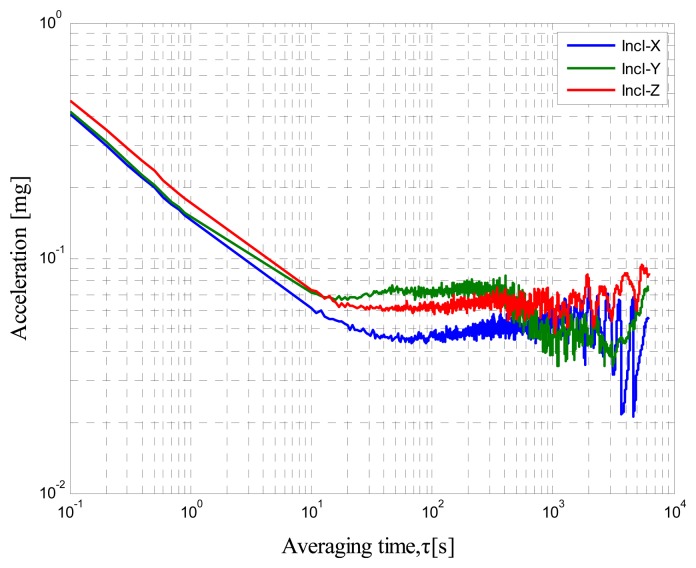
Inclinometer RAV results.

A self-made MIMU that consists of mutually orthogonal axis accelerometers, gyroscopes, and inclinometers is tested. The AV tests are conducted with a sampling rate of 250 Hz, room temperature of 25 °C, and total sampling time of 6 h to determine the minimum bias stability time of the output. The static data are collected by LabVIEW. The RAV results are shown in Figures 2, 3 and 4. Figure 2 shows the accelerometer data, Figure 3 shows the gyro data, and Figure 4 shows the inclinometer data.

These log–log figures show that steadying the sensor drift characteristic takes approximately 200 s, which means that accelerometer, gyro, and inclinometer biases are much noisier at less than 200 s. The bias instability term in the long cluster time becomes the lead error, whereas the random walk is the dominant error in the short period. This condition implies that the bias will not change severely over the 200 s intervals. Thus, the sensor should be averaged over a period of at least 200 s before data collection and the random error can be considered as white noise within 200 s.

### 2.2. Traditional Calibration Methods

The 12-position calibration method is a popular method in laboratory calibration. This method always requires special references, such as a high-precision, three-axis turntable, for alignment. The vital procedure involves setting the inertial system to be mounted on a leveled surface, with each sensitive axis of every sensor pointing alternately up and down to decrease systematic errors. The accuracy of this calibration depends on how well the axes are aligned with the vertical axes of the local level frame of the turntable. After a rigorous alignment between the MIMU and the turntable, the tested MIMU is rotated to the fixed positions programmed before the tests (Table 1). Each axis of the gyroscope or accelerometer points upward and downward alternately. The left axes in each position rotate 180° around the fixed axis where we can obtain 12 data groups. Based on the AV results, the MIMU should be maintained for 200 s to keep outputs steady. The biases, scale factors, and cross-coupling of the MIMU sensors can then be calculated through the least squares method:

(4) $$\bar{A_{a}}=\left[\begin{array}{cccccc} +g & -g & 0 & 0 & 0 & 0\\ 0 & 0 & +g & -g & 0 & 0\\ 0 & 0 & 0 & 0 & +g & -g\\ 1 & 1 & 1 & 1 & 1 & 1 \end{array}\right]$$

(5) $$U=\left[\begin{array}{cccccc} U_{x1} & U_{x2} & U_{x3} & U_{x4} & U_{x5} & U_{x6}\\ U_{y1} & U_{y2} & U_{y3} & U_{y4} & U_{y5} & U_{y6}\\ U_{z1} & U_{z2} & U_{z3} & U_{z4} & U_{z5} & U_{z6} \end{array}\right]$$

(6) $$U=\bar{K}\cdot {\bar{A}}_{a}$$

(7) $$\bar{K}=U\cdot {\bar{A}}_{a}^{T}\cdot {\left({\bar{A}}_{a}\cdot {\bar{A}}_{a}^{T}\right)}^{-1}$$

(8) $$\bar{K}=\left[\begin{array}{cccc} k_{xx} & k_{xy} & k_{xz} & U_{0x}\\ k_{yx} & k_{yy} & k_{yz} & U_{0y}\\ k_{zx} & k_{zy} & k_{zz} & U_{0z} \end{array}\right]=[K|U_{0}]$$

$\bar{A_{a}}$ is a matrix formed with the MIMU input, and *U* represents the output of a triad of sensors (e.g., accelerometers). *K̄* can be solved with the matrix, including the biases, scale factors, and non-orthogonality, through the least squares method. The diagonal elements of matrix *K* represent the scale factors formed as *S_j_*, and *U*_0_ represents the sensor bias matrix; the remaining elements measure non-orthogonality.

However, the Earth rate for almost all the MEMS gyroscopes is very weak and is, thus, drowned by noise. Thus, the 12-position calibration is insufficient. Another method called the angle rate method is required. The biases, scale factors, and non-orthogonalities of the gyroscopes can be estimated based on the same principle of the 12-position method by rotating the MIMU precisely to a set of known angles and comparing the outputs of the MIMU with the references inputs. Therefore, all the main parameters of the MIMU accelerometers and gyroscopes can be distinguished in navigation systems by combining the traditional static multi-position calibration and angle rate methods.

## 3. Fast Field Calibration via Multi-Position Calibration

### 3.1. Multi-Position Calibration Theory

The multi-position calibration method has emerged in recent years as a result of the great demands for short, inexpensive, and effectively equipped calibration. This method, which can calibrate the error parameters in the field, is based on traditional methods with little demand for the turntable and a short calibration time. Local gravity and Earth rotation are the sensor inputs without other disturbances. The norms of the accelerometer outputs in static conditions are the same as those under local gravity. For the gyros, the norms of the outputs are equal to the rotation rate provided by a single-axis turntable. The fundamental concepts of the multi-position calibration method are shown in Equations (9) and (10):

(9) $${g_{x}}^{2}+{g_{y}}^{2}+{g_{z}}^{2}=g^{2}(\cos^{2}\theta_{g}+\cos^{2}\gamma_{g}+\cos^{2}\varphi_{g})=g^{2}$$

(10) $${\omega_{rx}}^{2}+{\omega_{ry}}^{2}+{\omega_{rz}}^{2}={\omega_{r}}^{2}(\cos^{2}\theta_{\omega }+\cos^{2}\gamma_{\omega }+\cos^{2}\varphi_{\omega })={\omega_{r}}^{2}$$

where *g* is the local gravity; ω_r_ is the given rotational rate; θ_g_, γ_g_, φ_g_ are the angles between each axis of MIMU accelerometers; and θ_ω_, γ_ω_, φ_ω_ represent the angles between each axis of the MIMU gyros and the given rotational velocity. Combining Equations (1) and (2) for MEMS accelerometers, the scale factor matrix $S_{a}=\left[\begin{array}{ccc} S_{ax} & 0 & 0\\ 0 & S_{ay} & 0\\ 0 & 0 & S_{az} \end{array}\right]$, the bias matrix *b_a_*[*b_ax_* *b_ay_* *b_az_*] *^T^*, and the misaligment matrix $T_{a}=\left[\begin{array}{ccc} 1 & -\Delta_{yz} & \Delta_{zy}\\ \Delta_{zy} & 1 & -\Delta_{zx}\\ -\Delta_{xy} & \Delta_{yx} & 1 \end{array}\right]$, in which Δ_*ij*_ stands for the angle of the *i* and *j* axes, are expressed as follows:

(11) $$T_{a}\approx T_{az}\cdot T_{ay}\cdot T_{ax}$$

(12) $$T_{az}=\left[\begin{array}{ccc} \cos\Delta_{z} & -\sin\Delta_{z} & 0\\ \sin\Delta_{z} & \cos\Delta_{z} & 0\\ 0 & 0 & 1 \end{array}\right]$$

(13) $$T_{ay}=\left[\begin{array}{ccc} \cos\Delta_{y} & 0 & \sin\Delta_{y}\\ 0 & 1 & 0\\ -\sin\Delta_{y} & 0 & \cos\Delta_{y} \end{array}\right]$$

(14) $$T_{ax}=\left[\begin{array}{ccc} 1 & 0 & 0\\ 0 & \cos\Delta_{z} & -\sin\Delta_{z}\\ 0 & \sin\Delta_{z} & \cos\Delta_{z} \end{array}\right]$$

Given that the angle deviation is so small that the sin and cos can be replaced by the angle, Equation (15) is derived as follows:

(15) $$T_{a}\approx \left[\begin{array}{ccc} 1 & -\Delta_{z} & \Delta_{y}\\ \Delta_{z} & 1 & -\Delta_{x}\\ -\Delta_{y} & \Delta_{x} & 1 \end{array}\right]$$

Based on the inputs of matrix *G* = [*g* 0 0]*^T^*, Equation (16) can be obtained with the misalingment, scale factors, and biases. The solution path of the gyros is consistent with that of the accelerometers:

(16) [laxlaylaz]=[1+Sax00−Δyz1+Say0Δzy−Δzx1+Saz][g 0​ 0]T+[baxbaybaz]T

For MIMU accelerometers, fast field multi-position calibration is used to solve Equation (17) and corresponds with Equation (18), which solves gyro error parameters. Therefore, the use of nonlinear equations is crucial. To search for the optimal value, the improved numerical Powell algorithm is used in solving the nonlinear equations. All the unknown parameters can then be acquired:

(17) $$f(b_{a},S_{a},T_{a})=\text{argmin}\sum_{n=1}^{N}\left(a_{x}^{2}+a_{y}^{2}+a_{z}^{2}-g^{2}\right)$$

(18) $$f(b_{\omega },S_{\omega },T_{\omega })=\text{argmin}\sum_{n=1}^{N}\left(\omega_{x}^{2}+\omega_{y}^{2}+\omega_{z}^{2}-\omega_{0}^{2}\right)$$

### 3.2. Powell Algorithm

The Powell algorithm is a straightforward searching algorithm first proposed in 1964 by Powell to solve unconstrained optimization problems. The derivatives do not requrie calculation because the algorithm only needs to calculate the function values when the function is a continuous one; through constant improvement, the Powell algorithm has been used in a wide range of applications [25,26]. The Powell method of least squares is used to search for optimal parameters, and the conjugate equations in the iteration are generated step by step. This method is a type of conjugate direction method. It is a very effective approach to achieve the minimum value of one function that is not strict with the initial value. The convergence rate is fast, and its partial optimization ability is classical. The specific numerical solution is as follows:

(1) Suppose that the following are given: *n* arbitrary original elements at arm's length $x_{i}^{0}\in R^{n}(i=1\sim n,n=9)$, a group of linearly independent search directions *e*_0_ =(*e*_1_, …, *e_n_*), and permissible error ε <0.
(2) Begin the search process from the starting point along *e*_1_, …,*e_n_*:
  (19) $$f(x_{i})=\underset{\lambda }{\min}f(x^{i-1}+\lambda_{i}e_{i})$$
(3) Check whether the termination criteria are satisfied by taking the acceleration direction of Powell as *e_n_*:
  (20) $$e_{n}=\underset{1\leq i\leq n}{\max}\left|f(x_{i})-f(x_{0})\right|=\left|f(x_{big})-f(x_{0})\right|$$
  If ‖*e_n_*‖≤ ε, then the iterations end, and the optimal calibration results can be obtained. Otherwise, proceed to Step 4.
(4) To determine the search direction (*m*(0 ≤ *m* ≤ *n* − 1)) according to Equation (21), proceed to Step 5 if Equation (22) can be satisfied. Otherwise, proceed to Step 6.
  (21) $$f(x_{m})-f(x_{m+1})=\underset{0\leq j\leq n-1}{\max}\{f(x_{j})-f(x_{j+1})\}$$
  (22) $$f(x_{0})-f(x_{n})+f(2x_{n}-x_{0})=2\left[f(x_{m})-f(x_{m+1})\right]$$
(5) Adjust the search direction starting from point *x_n_* along the search direction of *e_n_* with the solution of λ*_n_* to satisfy Equation (23):
  (23) $$f(x_{n}+\lambda_{n}e_{n})=\underset{\lambda }{\min}f(x_{n}+\lambda_{n}e_{n})$$
  *x_k_*_+1_ = *x_n_* + λ*_n_e_n_*, *e_j_* = *e_j_* +1, *j* = *m*, *m*+1, …, *n* − 1, *k* =*k* +1; go back to Step 2 to continue.
(6) Without adjusting the search direction, set *x_k_*_+1_ = *x_n_*, *k* = *k*+1. Repeat Step 2 to continue this process.

The error parameters of the accelerometers and MIMU gyros can be solved in a relatively short time after the iterative process above. At least nine positions must be rotated in analyzing the fast field multi-position process involving an unknown number of parameters. However, additional positions are needed to obtain highly accurate calibration results given the correlation and useless position in the calibration process. The specific multi-position group is shown in Table 2. Each axis of every single sensor faces up or down in the first 12 positions. The MIMU is then positioned with the angle between different axes at 45° to generate six other positions or more. Unlike in traditional calibration methods, a high degree of alignment in each position is unnecessary in the proposed method.

The fast field calibration method based on the Powell algorithm is validated with synthetically generated data through MATLAB. The generated data are intended to represent actual data that would be collected from the MIMU. The actual acceleration vectors of the 18 positions can be determined by the decomposition of gravity in the accelerometer and inclinometer simulation. A constant angular rate, which plays the same role as gravity in accelerometers, is assumed for the gyroscopes. The assumptive acceleration vectors of these positions can be determined by the actual vectors and assumptive parameters of the MIMU. The objective function is then established, and the Powell algorithm is used to solve the nonlinear problem. The simulated parameters of the MIMU can be searched and compared with the assumptive parameters. The assumptive parameters and relative errors between the simulated results and assumptions are shown in Table 3. The relative errors are less than 1000 and show the effectiveness of the proposed algorithm.

## 4. Calibration Results of Different MIMU Methods

As all the calibration tests are performed at room temperature, temperature compensation is not considered. The main parameters of the tested MIMU are listed in Table 4, where the biases and random walks result in the AV condition.

**Figure 5.**
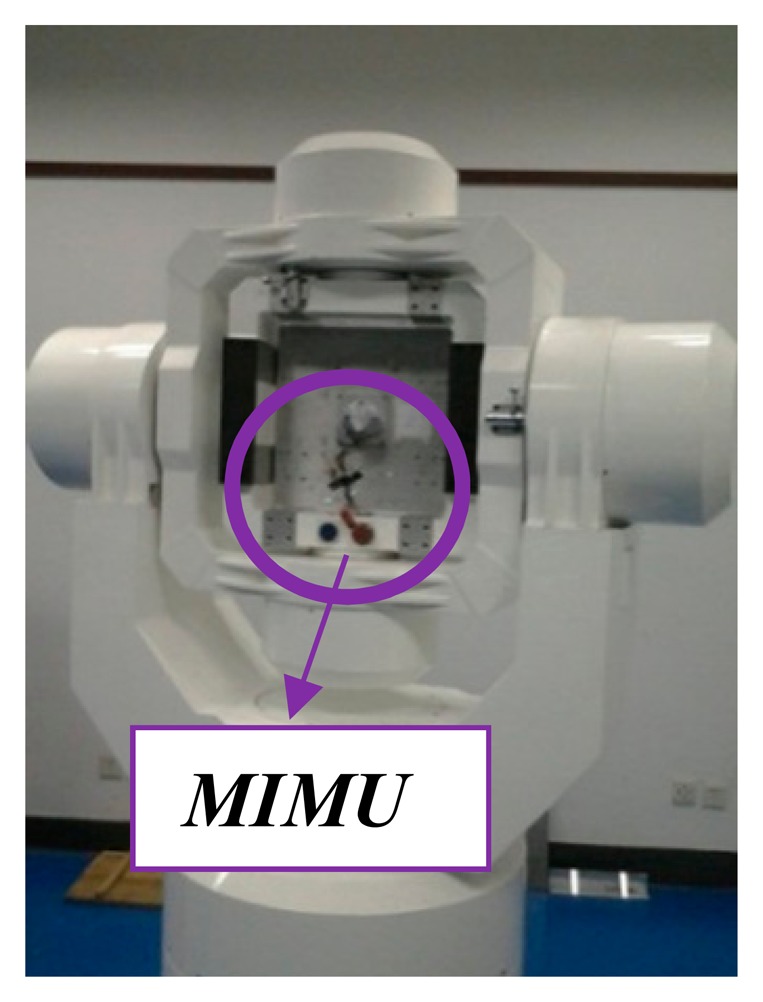
Three-axis turntable in laboratory calibration.

The tested MIMU is calibrated using traditional methods. The alignment requirements in these tests are high, and the initial alignment is the most important factor. Each inclinometer axis is set as the basis, which means that the inclinometer axes are aligned to the reference of the three-axis turntable shown in Figure 5. The data collection time at each position is based on the results of the AV tests. The calibration results after data processing are shown in Table 5.

Specific force and angular velocity should be equal to gravity and rotation rate, respectively, in terms of magnitude. The fast field multi-position calibration is performed according to the positions shown in Table 2 for the same MIMU. The static calibration for inclinometers and accelerometers can meet the condition of a multi-position calibration, but for the gyroscope, an additional rate must be provided by a single-axis turntable because the MEMS gyroscope is insensitive to the Earth's rotation rate. The single-axis turntable is very small, inexpensive, and portable and is, thus, suitable for field applications. The rotation rate of our single-axis turntable ranges from 0.1°/s to 20°/s. We select 10°/s in our test. The Powell method is used to search for the optimal error parameters by solving the nonlinear equations. The calibration results are also listed in Table 5.

Table 5 clearly indicates that the performance of the multi-position method in the judgment of biases and scale factors is comparable to that of traditional calibration methods. Considering the capitalized calibration cost, the fast field multi-position calibration method is proved to be practical.

## 5. Vehicle Test of Navigation Performance with Compensated MIMU Parameters

Comparing the calibrated sensor parameters of the different methods is not enough to investigate the calibrated results further. The navigation performance of these compensated parameters in field tests is thus analyzed. A decision is made in favor of real test drives against the calibrated measurement data to validate the stability and reliability of the proposed calibration method. This condition ensures realistic driving conditions, which include car vibrations, realistically scaled acceleration, and angular rate signals, for the used MIMU. The influence of temperature is excluded by using the car air conditioner to equalize the temperature inside the car and in the laboratory.

**Figure 6.**
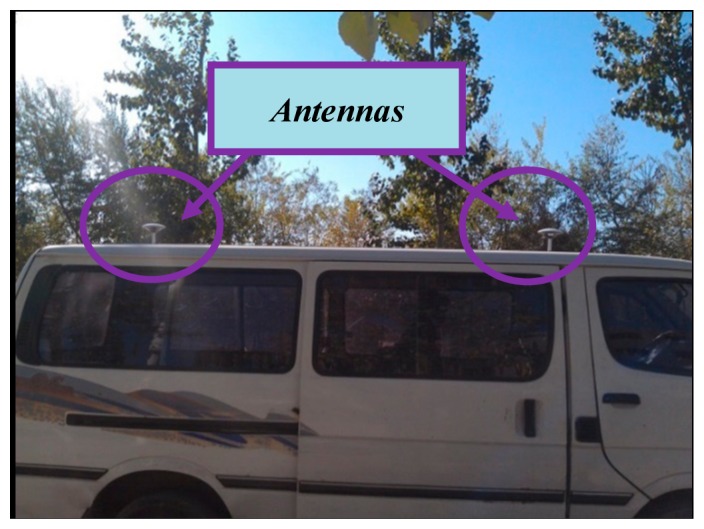
Vehicle test setup.

The tested MIMU is installed in the vehicle with a new GPS/INS device consisting of three optical fiber gyroscopes, three quartz accelerometers, and a RTK (Real-time kinematic) GPS with 2 cm + 1 ppm CEP (Circular Error Probable). The outputs serve as the standard to evaluate the navigation results. During the experiment, no relative motion must occur between the tested MIMU and the GPS/INS. The relative MIMU and GPS/INS positions are ignored when calculating the navigation results. Figure 6 shows that the GPS/INS antennas are placed on the vehicle roof to receive GPS signals, whereas the other experimental devices, such as the mobile power supply and laptop computers, are placed in the cargo hold.

**Figure 7.**
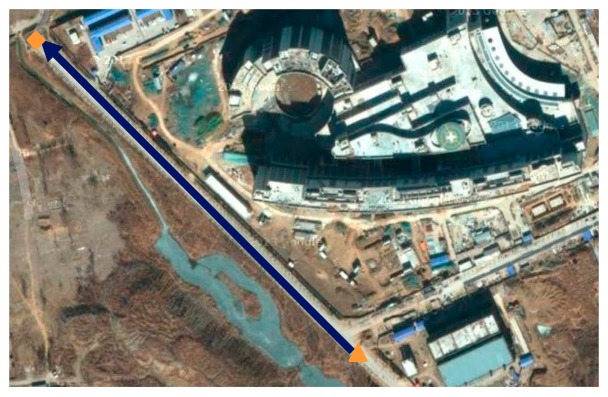
Straight loop. The blue line is the straight trajectory, the orange triangle is the zero point, the orange square is the end point, and the arrows show the running direction.

**Figure 8.**
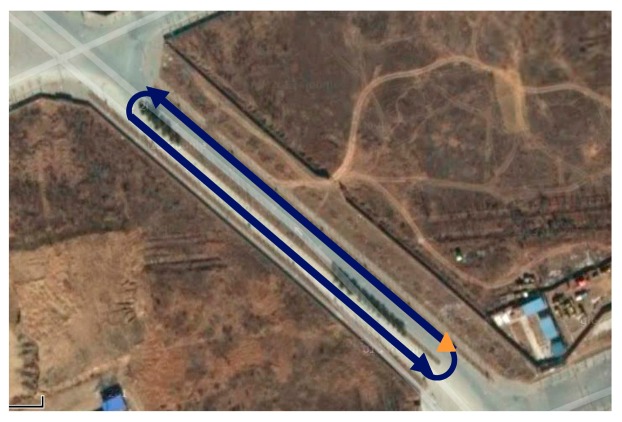
Circle loop. The blue line is the circle trajectory, the orange triangle is the zero and end points, and the arrows show the running direction.

The following two types of loops are desired: a trajectory that runs along a straight line and one that runs along a circle. The cargo for each loop is run five times (*i.e.*, two loops are shown in Figures 7 and 8). The vehicle test is performed in the Zhongguancun Life Science Park in the Chang-Ping District of Beijing. Similar to the calibration tests above, the MIMU should be maintained in a static state for 200 s in each run before data are collected. When the cargo reaches the destination, data acquisition should be stopped immediately.

Given that the time computed is non-identical in various sensors, maintaining the MIMU and GPS/INS in a one time period is important. Comparisons only make sense in this manner. Considering the real-time and accuracy of attitude update, this study utilizes the details of error compensation based on the theory of optimal twin-sample rotation vector navigation algorithm. The group data of each type of vehicle test trajectory are illustrated in the following figures (results resemble those of other groups). Given that the sky channel is divergent, this channel is set aside in the test.

**Figure 9.**
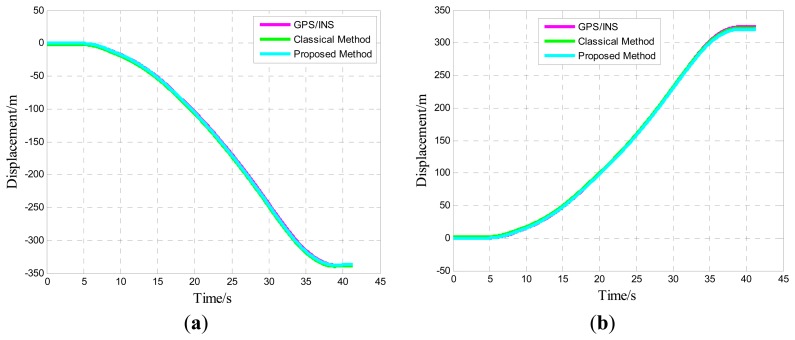
Time-varying position curve: (**a**) Eastward position; and (**b**) Northward position.

**Figure 10.**
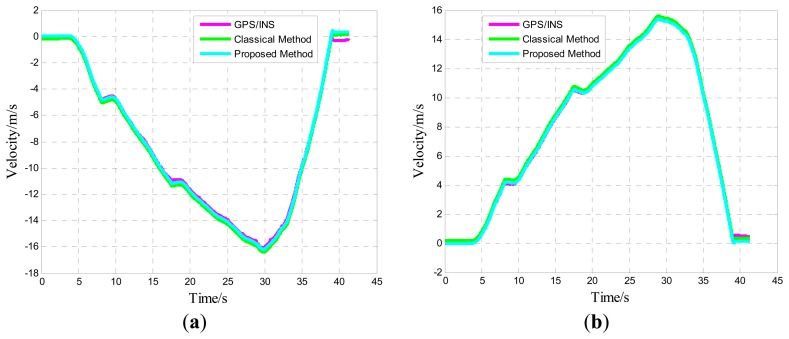
Time-varying velocity curve: (**a**) Eastward velocity; and (**b**) Northward velocity.

**Figure 11.**
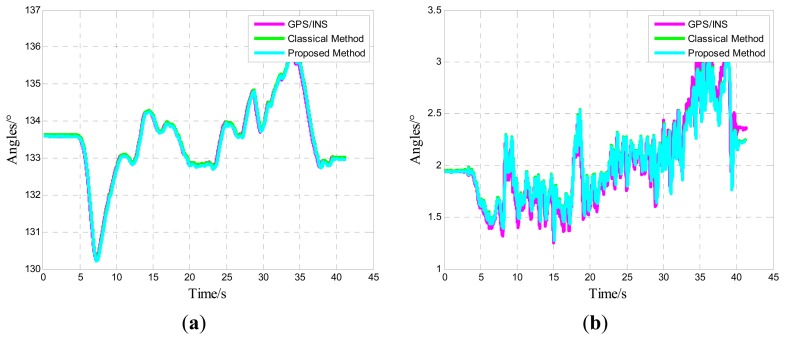

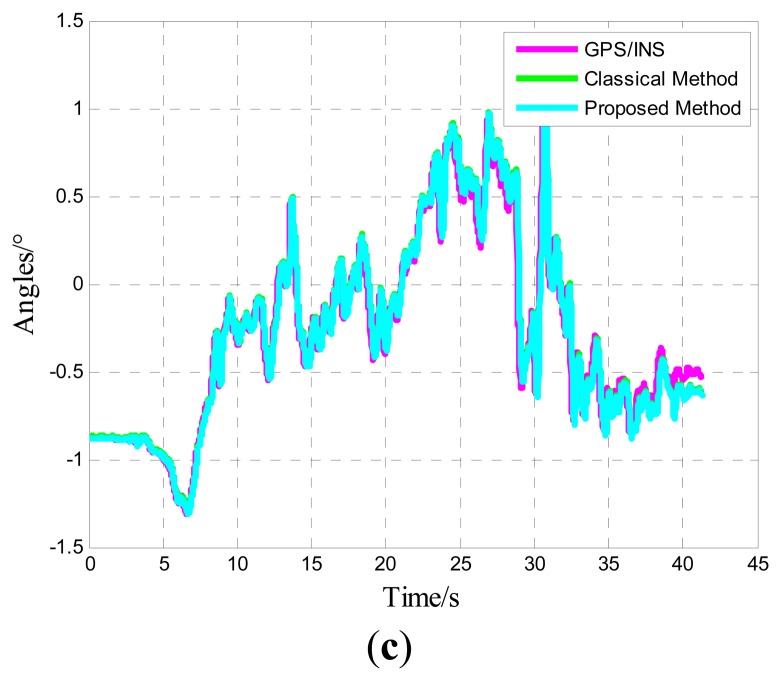
Time-varying angle curve: Yaw angle; (**b**) Pitch angle; and (**c**) Roll angle.

**Figure 12.**
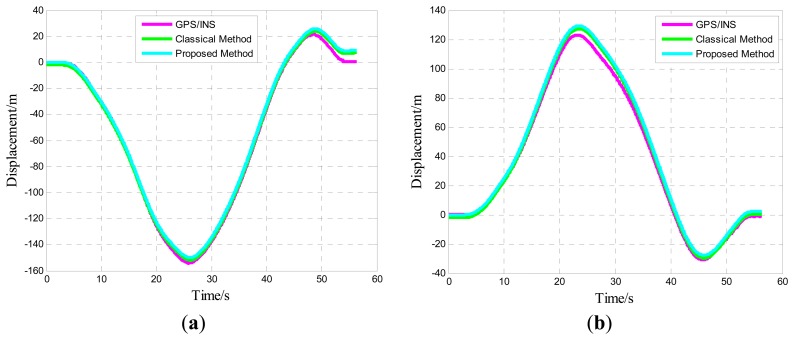
Time-varying position curve: (**a**) Eastward position; and (**b**) Northward position.

**Figure 13.**
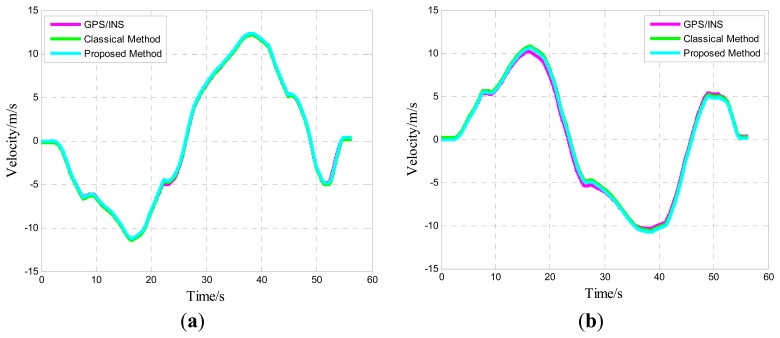
Time-varying velocity curve: (**a**) Eastward velocity; and (**b**) Northward velocity.

**Figure 14.**
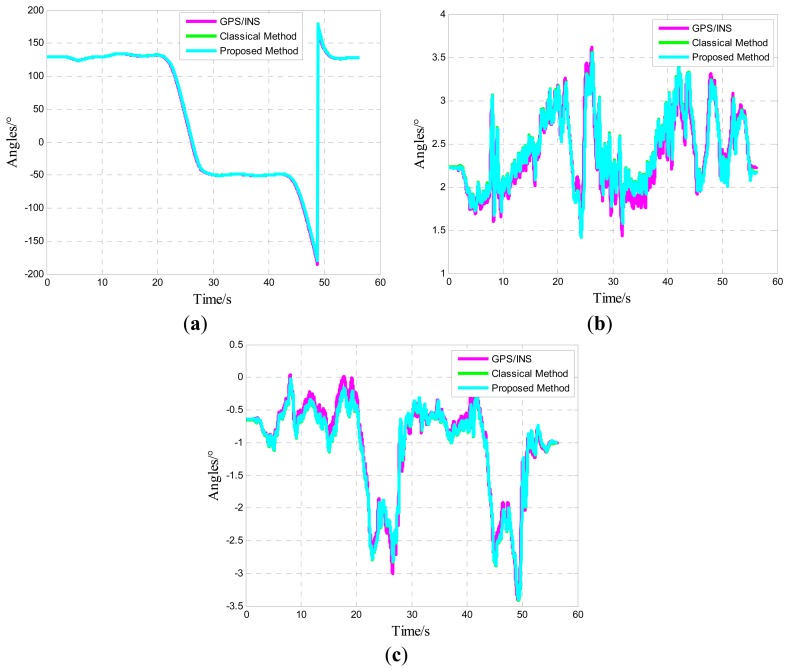
Time-varying angle curve: (**a**) Yaw angle; (**b**) Pitch angle; and (**c**) Roll angle.

One of five straight vehicle traces and navigation results is shown in Figures 9, 10 and 11. The navigation time of the straight traces is approximately 40 s. The eastward displacement is approximately 340 m, whereas the northward displacement is approximately 320 m. Similarly, one of five circle vehicle traces and navigation results is shown in Figures 12, 13 and 14. The navigation time of the circle traces is approximately 55 s. The maximum eastward displacement is approximately 150 m, whereas the maximum northward displacement is approximately 120 m. These graphs show that the angle deviation is small, whereas the maximum deviation of the position reaches up to approximately 10 m.

One of the five straight tracks is shown in the following:

Position information reflects all factors that affect the navigation precision, especially the errors of the MIMU. The comparison of the position information of the classical and proposed methods is presented in Table 6. A GPS/INS device provides “true ground” information, which is the trace reference. Navigation errors are calculated at the final point of each trajectory. Considering that the biases of gyroscopes and accelerometers are composed of constant and random parts and that the initial alignments are not absolutely the same, the position errors of different traces seem to be random. The absolute mean value could represent the overall performance. In the straight line tests, the eastward absolute mean values of the classical and proposed methods are 7.54 and 7.92 m, respectively. The northward absolute mean values are 5.33 and 6.27 m. The eastward absolute mean values in the circle loop tests are 11.39 and 12.36, whereas the northward absolute mean values are 3.59 m and 3.80 m. The navigation errors of the classical and proposed methods are generally close to each other. However, the navigation errors of the proposed method are slightly higher than those of the classical method, although the differences are acceptable.

## 6. Conclusions

The biases and scale factors of MEMS inertial sensors drift with time variation in micro inertial measurement units. This condition will have a definitive effect on navigation performance. Thus, calibration is essential in determining these error parameters. However, traditional calibration methods are time consuming, labor intensive, and inconvenient to use. In this work, we, thus, propose a new fast field self-calibration method that does not require precise calibration equipment and strict alignment accuracy of the rotational position. Based on the MIMU error model, the Powell method is used in solving nonlinear equations to determine the different types of error sources containing accelerometer and gyro biases, scale factors, and others. The tested MIMU is placed in a set of positions in the calibration procedure. The fast field calibration is much more convenient compared with traditional calibration methods, but calibration results are analogous. In special cases (e.g., lack of precise calibrated devices in a weapons launch site), the error parameters can be calibrated rapidly through fast field calibration to improve the navigation accuracy to some extent. Generally, calibration has comparatively important theoretical and practical values. However, this approach does not consider the temperature factor and fails to calibrate to a precise extent when the accelerometer and gyroscope triads are significantly misaligned. This issue can be explored further in future studies.

## Figures and Tables

**Table 1.** Gravity inputs applied in each micro inertial measurement unit (MIMU) position.

Position Number	Direction of MIMU Axes			Gravity Applied		
	
	*X*-axis	*Y*-axis	*Z*-axis	*X*-axis	*Y*-axis	*Z*-axis
1	Upward	East	North	*_g_*	0	0
2	Upward	West	South	*_g_*	0	0
3	East	Upward	North	0	*g*	0
4	West	Upward	South	0	*g*	0
5	East	North	Upward	0	0	*g*
6	West	South	Upward	0	0	*g*
7	Downward	East	North	−*g*	0	0
8	Downward	West	South	−*g*	0	0
9	East	Downward	North	0	−*g*	0
10	West	Downward	South	0	−*g*	0
11	East	North	Downward	0	0	−*g*
12	West	South	Downward	0	0	−*g*

**Table 2.** Eighteen positions for calibrating the MIMU.

**Position No.**	**1**	**2**	**3**	**4**	**5**	**6**
**Illustration**	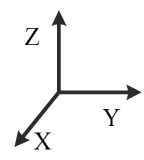	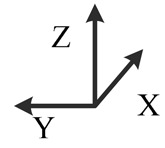	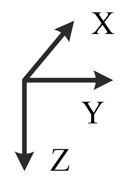	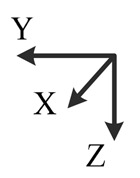	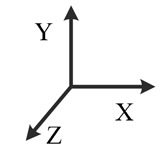	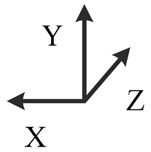
**Description**	*z*-upward, *x*-south, *y*-east	*z*-upward, *x*-north, *y*-west	*z*-downward, *x*-north, *y*-east	*z*-downward, *x*-north, *y*-west	*y*-upward, *x*-east, *z*-south	*y*-upward, *x*-west, *z*-north
**Position No.**	**7**	**8**	**9**	**10**	**11**	**12**
**Illustration**	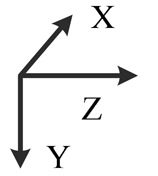	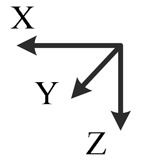	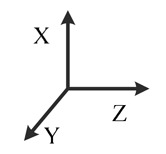	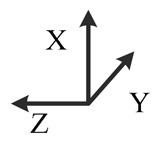	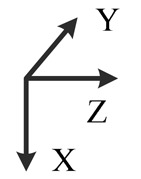	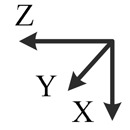
**Description**	*y*-downward, *x*-east, *z*-north	*y*-downward, *x*-west, *z*-south	*x*-upward, *y*-south, *z*-east	*x*-upward, *y*-north, *z*-west	*x*-downward, *y*-north, *z*-east	*x*-downward, *y*-south, *z*-west
**Position No.**	**13**	**14**	**15**	**16**	**17**	**18**
**Illustration**	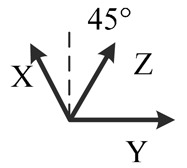	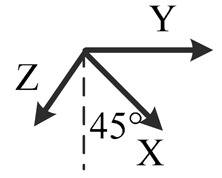	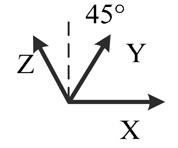	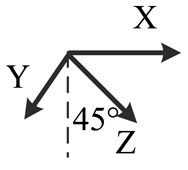	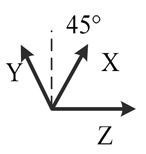	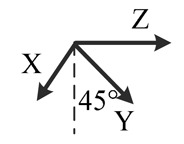
**Description**	*y*-east, *z*-north-upward with 45° pitch, *x*-south-upward with 45° pitch	*y*-east, *z*-south-downward with 45° pitch, *x*-north-downward with 45° pitch	*x*-east, *y*-north-upward with 45° pitch, *z*-south-upward with 45° pitch	*x*-east, *y*-south-downward with 45° pitch, *z*-north-downward with 45° pitch	*z*-east, *x*-north-upward with 45° pitch, *y*-south-upward with 45° pitch	*z*-east, *x*-south-downward with 45° pitch, *y*-north-downward with 45° pitch

**Table 3.** Assumption of the main parameters and simulation errors of the MIMU.

Sensors	Axis	Biases		Scale Factors	
	
		Assumption	Simulation Errors	Assumption	Simulation Errors
Gyroscope (°/s)	*X*	0.0100	1.706 × 10^−4^	1.0001	0.796 × 10^−4^
	*Y*	0.0200	0.854 × 10^−4^	1.0002	0.190 × 10^−4^
	*Z*	0.0300	0.903 × 10^−4^	1.0003	0.984 × 10^−4^
Accelerometer (mg)	*X*	1.0000	1.419 × 10^−4^	1.0001	0.044 × 10^−4^
	*Y*	2.0000	0.218 × 10^−4^	1.0002	0.853 × 10^−4^
	*Z*	3.0000	2.922 × 10^−4^	1.0003	0.238 × 10^−4^
Inclinometer (mg)	*X*	−1.0000	0.357 × 10^−4^	0.9991	0.513 × 10^−4^
	*Y*	−2.0000	0.891 × 10^−4^	0.9992	0.943 × 10^−4^
	*Z*	−3.0000	1.921 × 10^−4^	0.9993	0.475 × 10^−4^

**Table 4.** Main parameters of the tested MIMU.

Parameter	MIMU
**Gyroscope Full Scale**	±400°/s
**Gyroscope Bias (1σ)**	0.5°/h
**Gyroscope ARW**	$0.15^{\circ }\sqrt{hr}$
**Accelerometer Full Scale**	±10*mg*
**Accelerometer Bias (1σ)**	0.05*mg*
**Accelerometer ARW**	$0.06m/s/\sqrt{hr}$
**Inclinometer Full Scale**	±1.7*g*
**Inclinometer Bias (1σ)**	0.06*mg*
**Inclinometer ARW**	$0.08m/s/\sqrt{hr}$

**Table 5.** Calibration results of different methods.

Sensors	Axis	Calibrated Results			

		Traditional Method		Multi-Position Method	
	
		Biases	Scale Factors (Unitless)	Biases	Scale Factors (Unitless)
Gyroscope (°/s)	*X*	0.002965	1.0010	0.001954	1.0015
	*Y*	−0.03110	1.0009	−0.03172	1.0014
	*Z*	−0.04269	1.0007	−0.04389	1.0009

Accelerometer (*mg*)	*X*	5.0000	1.0004	5.232	1.0005
	*Y*	0.7574	1.0004	1.1271	1.0005
	*Z*	−0.2963	1.0007	−0.4389	1.0008

Inclinometer (*mg*)	*X*	0.4637	0.9975	0.6008	0.9984
	*Y*	−0.3117	0.9983	−0.5071	0.9981
	*Z*	−0.3125	0.9976	−0.4136	0.9981

**Table 6.** Comparison of position errors of the classical and proposed compensation method in vehicle tests.

Trace	Type	Run No.	True Ground (GPS/INS)	Navigation Errors by Classical Compensation	Navigation Errors by Proposed Compensation
**Straight Line**	**East (m)**	1	−337.83	−4.92	−3.55
		2	−338.67	12.98	15.94
		3	−343.46	6.14	−1.13
		4	−341.29	10.32	12.39
		5	−340.12	−3.34	6.57

		**Absolute Mean**		**7.54**	**7.92**

	**North (m)**	1	324.61	1.27	2.61
		2	325.37	9.43	12.93
		3	323.48	3.94	3.89
		4	318.50	4.59	6.12
		5	320.74	7.45	5.83

		**Absolute Mean**		**5.33**	**6.27**

**Circle Loop**	**East (m)**	1	−2.79	−8.41	−16.26
		2	0.53	−9.03	−12.71
		3	0.14	8.73	9.02
		4	−1.94	15.57	13.39
		5	1.45	−11.75	−10.44

		**Absolute Mean**		**11.39**	**12.36**

	**North (m)**	1	2.24	−2.14	−1.91
		2	−0.99	3.87	−4.60
		3	−0.83	0.95	2.31
		4	1.27	4.60	3.04
		5	3.04	−6.39	7.12

		**Absolute Mean**		**3.59**	**3.80**
